# An economic perspective on personalized medicine

**DOI:** 10.1186/1877-6566-7-1

**Published:** 2013-04-19

**Authors:** Sairamesh Jakka, Michael Rossbach

**Affiliations:** 360 Fresh Inc, 3600 West Bayshore Road, Suite 102, Palo Alto, CA 94303 USA; Office of Business Development, Genome Institute of Singapore, 60 Biopolis Street, 138672 Singapore, Singapore

**Keywords:** Clinical practice, Companion & molecular diagnostics, Healthcare, Incentives, Personalized medicine, Translational genomics

## Abstract

The concept of personalized medicine not only promises to enhance the life of patients and increase the quality of clinical practice and targeted care pathways, but also to lower overall healthcare costs through early-detection, prevention, accurate risk assessments and efficiencies in care delivery. Current inefficiencies are widely regarded as substantial enough to have a significant impact on the economies of major nations like the US and China, and, therefore the world economy. A recent OECD report estimates healthcare expenditure for some of the developed western and eastern nations to be anywhere from 10% to 18%, and growing (with the US at the highest). Personalized medicine aims to use state-of-the-art genomic technologies, rich medical record data, tissue and blood banks and clinical knowledge that will allow clinicians and payors to tailor treatments to individuals, thereby greatly reducing the costs of ineffective therapies incurred through the current trial and error clinical paradigm. Pivotal to the field are drugs that have been designed to target a specific molecular pathway that has gone wrong and results in a diseased condition and the diagnostic tests that allow clinicians to separate responders from non-responders. However, the truly personalized approach in medicine faces two major problems: complex biology and complex economics; the pathways involved in diseases are quite often not well understood, and most targeted drugs are very expensive. As a result of all current efforts to translate the concepts of personalized healthcare into the clinic, personalized medicine becomes participatory and this implies patient decisions about their own health. Such a new paradigm requires powerful tools to handle significant amounts of personal information with the approach to be known as “P4 medicine”, that is *predictive, preventive, personalized* and *participatory*. P4 medicine promises to increase the quality of clinical care and treatments and will ultimately save costs. The greatest challenges are economic, not scientific.

**Electronic supplementary material:**

The online version of this article (doi:10.1186/1877-6566-7-1) contains supplementary material, which is available to authorized users.

## Personalized medicine

**Figure 1:**
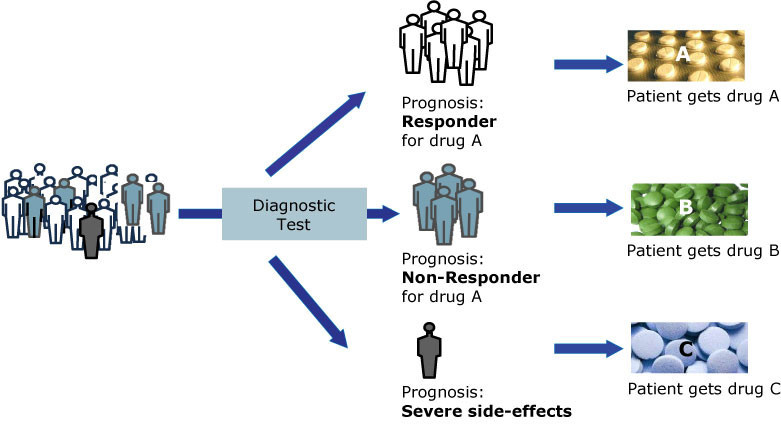
**Better patient treatments through advanced diagnostics and personalized medicine.** Diagnostic tests will guide the clinical decision-making to prescribe a specific drug, depending on the patient’s prognosis to be a responder or non-responder to a given medication (VFA Bio).

Avoiding trial and error phases through better and truly personalized medicine, using genetic profiles to identify the best possible drug and therapy for a given patient and reducing adverse effects, are among the key personalized medicine goals that will benefit not only patients, but healthcare systems in general. The P4 medical approach, *predictive, preventive, personalized* and *participatory* medicine, will help to identify the right drug for the right patient at the right time, avoiding the prescription of costly and ineffective drugs and preventing potentially harmful side-effects (Figure 1). In this regard, P4 medicine describes a systems approach to medicine that includes the aforementioned four aspects and several factors fuel this development, *viz.* (i) the appreciation that medicine is a knowledge-based, information science, (ii) systems approaches are inextricably linked with studying the tremendous complexity of diseases and disease analysis, (iii) new computational and mathematical methods will allow for the analysis of thousands of data points associated with each individual patient. Particularly, novel genome-based diagnostic technologies represent a significant advance in medical practice in comparison with the current prevention methods; a combination of genetic knowledge and clinical studies is expected to impart a significant advance toward preventive medicine and, subsequently, prospective medicine. Perhaps the biggest hurdle in this regard is that the developers of novel molecular diagnostics are not well aligned with the pharmaceutical manufacturers whose products might be affected by the knowledge gained from the diagnostics. We are in a great need of much broader and better collaborations between the diagnostic test developers, who are focusing on the targeting early drug development efforts and the pharmaceutical companies that manufacture and sell the drugs.

Rapid advances in electronic medical record systems utilized by patient care providers is showing an enormous opportunity to deliver efficiencies in retrieving, updating and providing real time access to individualized patient records in a wide variety of circumstances (battlefields, travel situations, emergency rooms, etc.). Such real-time medical record systems will provide state of the art data about patient responsiveness to therapies in specific disease states (including up to date information on therapeutics and treatments by responder category), and enable cost-effective treatments and personalized care pathways.

In a third category, biomarker-based diagnostics have a significant impact on the development of both therapeutic and diagnostic agents, a concept known as *theranostics* and are highly relevant for drug development and future personalized medicine. At present, one can identify several different models for such future medicines, *viz.* (i) drugs for a certain phenotype, (ii) drugs for a very small and specific patient population, and (iii) one drug for one patient, which is the classic and original idea of personalized medicine, implying the development of a targeted therapy for each and every individual patient. For all models, the overall personalized medicine structure needs a closer coordination among researchers, clinicians, manufacturers, and payors. P4 medicine could mature as the idea of consumer-driven healthcare advances with a strong focus on individual health needs, and on concentrating more on preventive care, rather than on treatments and resultant expenditures once a disease has emerged.

The new theranostics paradigm is the key to truly private and personalized medicine, a tailored approach to a patient’s treatment and outcome based on the molecular analysis of the genome. This approach attracted a lot of attention in the biomedical and clinical field and generated much excitement in recent years; however, so far, only a few personalized medicine based diagnostic tests have achieved high levels of clinical adoption. It is estimated that nearly 30-40% of patients get ineffective drugs (where the costs arising from adverse events and toxicities outweigh the benefits). Theranostics can cut down the false-positive rates tremendously and improve upon targeted therapy. Currently 20% of the Pharma R&D is gene-based, and this is increasingly showing promise in identifying novel therapies at a fraction of the costs of the complex clinical trials processes.

## Aligning incentives? Challenges!

*En route* to clinical practice of personalized medicine, several challenges remain, among them scientific ones, i.e. a poor understanding of the underlying pathways and molecular networks in various, particularly complex diseases, and a lack of biomarkers associated with some diseases and practical ones, a lack of the tools that could speed the mining of reams of data for clinically significant meaning, and the lack of access to current tools to access and utilize existing data (Garcia Martinez de Lecea, 2012).

Although the challenges in each of these areas are significant, a consistent factor among all three is that the operational and economic challenges seem to be the major hindrances. Often, operational challenges can be resolved within a particular stakeholder group, whereas poorly aligned stakeholder incentives differing economic benefits to efficiency incentives are more complex and more difficult to be resolved. For example, a medical treatment paradigm that rewards clinicians (in terms of economic gain) for foregoing medical tests the same time outcomes are typically enhanced by greater testing, is solved the moment that costs for testing or technologies that limit the need for testing in certain subpopulations is introduced. In contrast to the promise of personalized healthcare to dramatically reduce costs, most payers have been slow in investing in personalized healthcare. Reasons are numerous, and include the following: (i) it is difficult to identify, which diagnostic tests, corresponding assays, information technologies and operational systems will truly save costs; (ii) although individual diagnostic tests or systems may not be very expensive, the overall costs could be amazingly high; (iii) data security is a very challenging task as private information must be protected, particularly in the stages of investigation and development; (iv) establishing standards in healthcare has always been very challenging; and (v) no structures exist that allow payers to assess cost savings from prognostic and preventive diagnostic testing. However, diagnostic tests tailored to a patient’s conditions, comorbidities, medical record information, including past history of medications may help to avoid expensive therapies, such as chemotherapies in cancer treatment, and significantly minimize costly adverse procedures. Companion diagnostic assays for Herceptin treatment is a good example of the value provided in predictive tests prior to prescribing the treatment regimen. As such, novel diagnostics and healthcare information technology (Health-IT) tools and systems have the potential of being cost effective for payers and providers. In contrast, diagnostics that save only small amounts per patient or have a low probability of identifying patients requiring intervention are not very cost effective.

Furthermore, the high customer turnover of many commercial payers makes it less attractive for payers to reimburse prophylactic tests that minimize the likelihood of a disease occurring much later in life. Also, to differentiate between a diagnostic test that actually saves costs on a long-term basis and tests that create costs, it may be in the interests of payers to delay adopting novel biomarker-based diagnostics since actual cost savings of such tests may not be known until a test has been on the market for some time. Consequently, the generation of high-quality health economics evidence could provide a basis and the confidence that enables payers to faster adopt diagnostic tests and would align physicians’ incentives with patient care, clinical practice and outcomes. In this sense, such developments could create a source of competitive advantage for payers that become better at identifying and implementing policies to promote cost-saving novel diagnostics. Another challenge is the current procedure-based reimbursement system for providers in many countries; physicians could be more likely to perform tests that increase the number of procedures performed than tests that reduce the number of procedures due to their financial interests. Also, diagnostic tests could identify a lot more patients of a certain risk for a specific disease, like cancer, which would align very well with economic interests of oncologists, for instance, whereas other tests may be cost neutral or have microeconomic disincentives.

**Figure 2:**
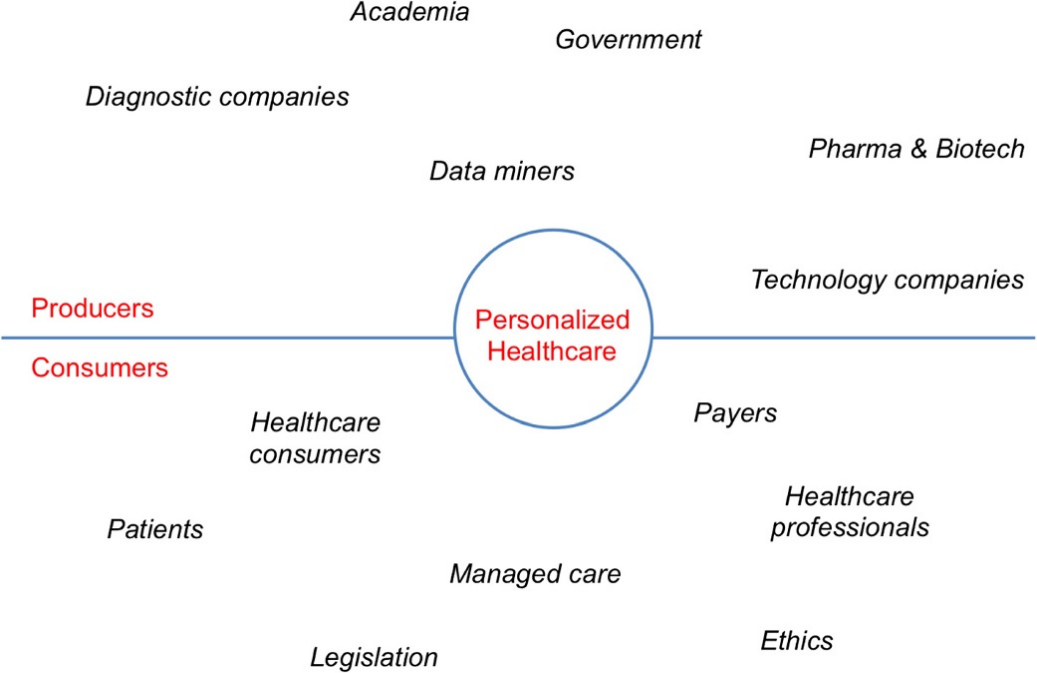
**Personalized healthcare, stakeholder space.**

In the future, a patient will be surrounded by a huge amount of data points that uniquely define the individual medical history and will reflect the current health status (Figure 2).

Mining such data will help to generate computational methods and algorithms to predict future clinical needs for each patient and to generate comprehensive profiles of patient groups. Such stratified patient populations and comprehensive systems biology approaches will result in powerful new diagnostics and therapeutics and provide invaluable new insights into prevention. Key to these developments will be the integration of medical data in the context of the dynamic biological pathways and molecular networks in both health and disease that are both actionable and predictive and thus useful to both clinicians and patients.

## Economic considerations

Generally speaking, the challenge is to find the balance between the patient’s benefit, economic value and clinical merit for biomarker-based diagnostics. Pharmaceutical companies are beginning to focus more on such biomarker-based diagnostics that come along with companion diagnostic tests that identify a patient’s likelihood of responding to a drug or experiencing side effects (toxicities) and are intended to assist physicians in making treatment decisions for their patients. The two main groups of companion diagnostics include (i) tests that have been developed after a drug has come to market and (ii) tests that are being developed in conjunction, as a companion, to the drug. Today, a majority of drugs in developmental pipeline come along with associated biomarker programs, with the number likely to be increasing. Such companion diagnostic tests can improve research productivity by decreasing trial sizes, increasing the speed to market and supporting higher drug prices. Companion diagnostics have the potential to significantly influence drug development and the commercialization of lead candidates through safer drugs with enhanced therapeutic efficacy. Today, knowledge about the molecular mechanisms and pathways a drug interferes with gained through next-generation genomic technologies is crucial for drug developers before clinical symptoms are studied in clinical trials. Such genomic technologies identify biomarkers that are qualified to be used as diagnostic and prognostic markers in certain diseases. In oncology, genome-based diagnostics are rapidly evolving as many pharmaceutical companies focus on the development of targeted therapies and consider the benefits for a diagnostic test to pair with a specific treatment. Such tests are showing potential in reducing tremendously the costs of clinical trials (close to 2/3rds of the clinical trial costs in some cases). A recent report estimates over 130 million in savings for pharma companies per approved compound. Unfortunately, scientific and clinical factors place limits on the pace of such developments. For many pathophysiological conditions, the underlying principles are far from being understood or the current scientific knowledge is insufficient to select for specific biomarkers at early stages of a disease. In other areas, there is no immediate clinical need for companion diagnostics. It seems that, in general, the potential to generate a greater value after market launch, through increasing market shares, is much more important for the economics of pharmaceutical and biomedical companies than making development more productive, and companion diagnostics may not contribute a lot to improve development productivity. Actually, they might even increase overall costs and delay drug developments since clinical trials must frequently be larger when companion diagnostics are employed and candidate biomarkers must be tested if it is unclear, which markers will be predictive. Further, regulatory authorities require marker-negative patients to be included in clinical trials. Moreover, decision analysis and an increased understanding of how humans make decisions (see generally Kahneman, Thinking Fast and Slow (Kahneman 2011) and the identification of the “cognitive biases” (unconscious distortions in reasoning that affect the rationality of decision making) it seems increasingly understood that the neutralization, quantification of data and examination through neutral mechanisms will greatly increase the correct decisions in any system, but especially in those that are extremely data laden and complex, like health and patient care.

Nonetheless, companion diagnostics and software, data analytics and other information technology advancements all have the potential to create a significant commercial benefit in markets with pricing flexibility in terms of market share, but are likely to be of higher value for later-to-market entrants. This is due to the fact that companion diagnostics and corresponding theranostic assays divide the market of treatable patients into groups and clusters, thus reducing market share. A companion drug, however, that is capable of identifying a group of patients that responds to a specific therapy very well, enables higher pricing and thus generates value to stakeholders. In this regard, the key is the payer’s price sensitivity that varies a lot by disease area; therefore, drug classes can be segmented according to their scientific and commercial potential.

Pharmaceutical companies may be more likely to invest in diagnostics and technologies that impact larger groups, generally such as those in areas like infectious diseases, immunology and oncology, with the latter being the most advanced field for personalized medicine. The segmentation also reveals disease areas where incentives are not aligned to drive investment, despite technical feasibility and clinical need; among them are antipsychotics or anticoagulants. Firms focusing on diagnostic tests provide a huge variety of different systems, among them recurrence and monitoring tests in cancer medicine, early-stage diagnostics, susceptibility tests for adverse or toxic side-effects of drugs, genomic tests for risk assessments of a particular inherited disease as well as tests that include companion diagnostics. Amid the excitement and attention paid to personalized cancer treatment, it is crucial to remember that other conditions like psychiatric disorders carry as great a societal burden, yet remain too poorly understood to benefit.

From an economic view, revenue generation with diagnostic tests remains challenging; albeit diagnostic tests influence most clinical decision-making, they only account for a small percentage of today’s health insurance expenditures.

However, P4 medicine indeed has a great potential to catalyze changes in the increasing costs of medical care and will ultimately result in reduced costs to the point where P4 medicine will be exported to the developing world; as such, P4 medicine will be the foundation of global health care in the future. The reduction of costs will be achieved through a variety of factors, among them is the digitalization of health care and advances in health care IT, novel translational technologies such as next-generation sequencing technologies entering clinical laboratories, and the emerging field of single-cell omics that allow the analysis of thousands of cells in a high throughput manner (Hood and Galas 2003). The central challenge of modern medicine is the stratification of patients, e.g. based on novel biomarkers, and the classification of patients into subgroups with different combinations of disease-perturbed networks (Ivshina 2006). Crucially, the adoption of the P4 concept will enable the focus of medicine to shift from disease to wellness, with enormous attendant cost savings to society resulting in a lower requirement for sick leave and a concurrent increase in productivity. Furthermore, many factors will converge to bring the costs of health care down in a striking manner so that the benefits of P4 medicine can be shared by rich and poor nations alike.

## Molecular diagnostics

Molecular diagnostics are a very attractive market segment to target due to the potential for significantly higher prices and gross margins. Companies like Myriad, Genomic Health or PathGen Diagnostics have developed innovative molecular diagnostics despite the risk that comes along with such developments in terms of development, approval, rates of provider adoption, sales prices or time-to-payer coverage. Since these risks are often underestimated, the (micro-) economics of molecular diagnostic companies that launched products vary significantly. The most important factors in this market are the rate of payer adoption and the time that is needed for approval. In terms of approval by the FDA, a 510 (k) approval may be sufficient for diagnostics that are prognostic indicators. Though, if a test directly influences clinical decision-making to go for a specific therapy, the FDA very likely will ask for a premarket approval that further increases the time to market significantly. In the present market and regulatory environment, molecular diagnostic companies therefore face challenging economics. However, as personalized medicine is progressing and evolving fast, more diagnostic tests will become available with developers, clinicians, patients, regulators and payers gaining more experience in this field. This in turn will likely influence and speed up the approval processes, and introduce better transparency.

As molecular diagnostics data begin to accumulate from next-generation sequencing / whole-genome sequencing efforts, for instance from tumor samples, disease classifications are likely to become even more precise and be extended into more diverse cancer types. Here, P4 medicine will clearly provide the technical and computational tools for more accurate patient stratifications. The developments in the molecular diagnostic sector will directly impact clinical trial structures and patient recruitments and will therefore induce a fundamental change in the business plans of the pharmaceutical industry.

From an economics perspective, the main mediators for personalized and private medicine are investments by biotech and pharmaceutical industries, which should take a long-term view on such investments. There is a trend towards outcome- and value-based pricing and reimbursement models in many countries and this greatly increases the financial value of P4 medicine, and particularly the incentives to invest in it. For reimbursement models, innovative strategies are required. For instance, reimbursements could depend on patient outcomes or risk-sharing models for drug and diagnostic coverage. The first step towards such healthcare systems is, however, that regulatory institutions improve both efficiency and clarity of approval processes for drugs and companion diagnostics. Also, the regulations allowing personalized medicine-based diagnostic test results to guide therapeutic interventions and therapy planning must be defined. Diagnostics and theranostics firms should demonstrate their willingness to comply with novel guidelines and even help shaping them – as a result they might justify higher prices due to the approval processes. From the authorities, however, stringent and fast approval processes are required, thus avoiding any negative impact on investments in the development of novel diagnostics and therapeutics. Examples would be a guideline that clinical data on marker-negative patients are not necessarily required, thus saving costs on the development side, or to allow the approval of companion diagnostics on the basis of retrospective trials of a novel diagnostic marker on, for instance, paraffin-embedded formalin-fixed samples. Diagnostic tests might make further treatments of a patient unnecessary and thus, physicians often have a higher interest in performing procedures rather than diagnostic tests.

The P4 approach will be successful for all parties involved: patients receive better care and physicians gain valuable information about the molecular characteristics of their patients’ diseases. The challenge is now to get P4 medicine into reality. Consequently, outcome-based approaches for reimbursement and joint approaches by payers and diagnostic firms will enable a faster adoption of personalized medicine-based diagnostics and thus improve the overall process and transparency of coverage decisions.

## Review, Conclusions

The developments in genomic technologies and near real-time access to medical records will enable the creation of more and more truly personalized medicine-based diagnostic tests and system-wide efficiencies in health care. To make P4 medicine a standard in clinical practice, all stakeholders should closely collaborate to reshape the incentives in personalized medicines and thus provide a basis for better and more efficient patient treatments and care pathways. Health systems reforms that promote a more flexible and value-based reimbursement for innovative diagnostics and therapeutics are critical to create stronger economic incentives for the development of personalized medicine and better care to patients.

P4 medicine will catalyze a transformation of standard care that promises to deal with the heretofore-impenetrable barriers of complexities of disease pathways through systems approaches, emerging technologies and powerful genome analytical tools. The promise is that the focus of medicine will be shifted from disease to wellness and that thousands of data points for each individual patient will be taken into account and define with exquisite specificity the nature of their wellness and any transitions into disease. Central to this view is the idea that the molecular, cellular, genomic and phenotypic data of eventually thousands of patients will be available for complex systems analyses, i.e. integration, normalizing, query, mining, analysis, storage and protection of data. This will catalyze the development of predictive and actionable models. The availability of these data will be necessary to exploit the infinite potential of the P4 medicine of the future.

The scientific, economic, and also the societal barriers for these objectives are considerable; overcoming the hurdles will require new ways for scientists to engage with each other, new relations between patients, scientists, and industry and, finally, will require new strategic partnerships among all stakeholders in the personalized medicine field.

## Consent

Written informed consent was obtained from the patient for publication of this report and any accompanying images.

## Authors’ original submitted files for images

Below are the links to the authors’ original submitted files for images. Authors’ original file for figure 1 Authors’ original file for figure 2
