# Supplementary figures and images for: An economic perspective on personalized medicine
Source: Hugo J. 2013 Apr 19;7(1):1. doi: 10.1186/1877-6566-7-1 (PMC4685168; doi:10.1186/1877-6566-7-1)

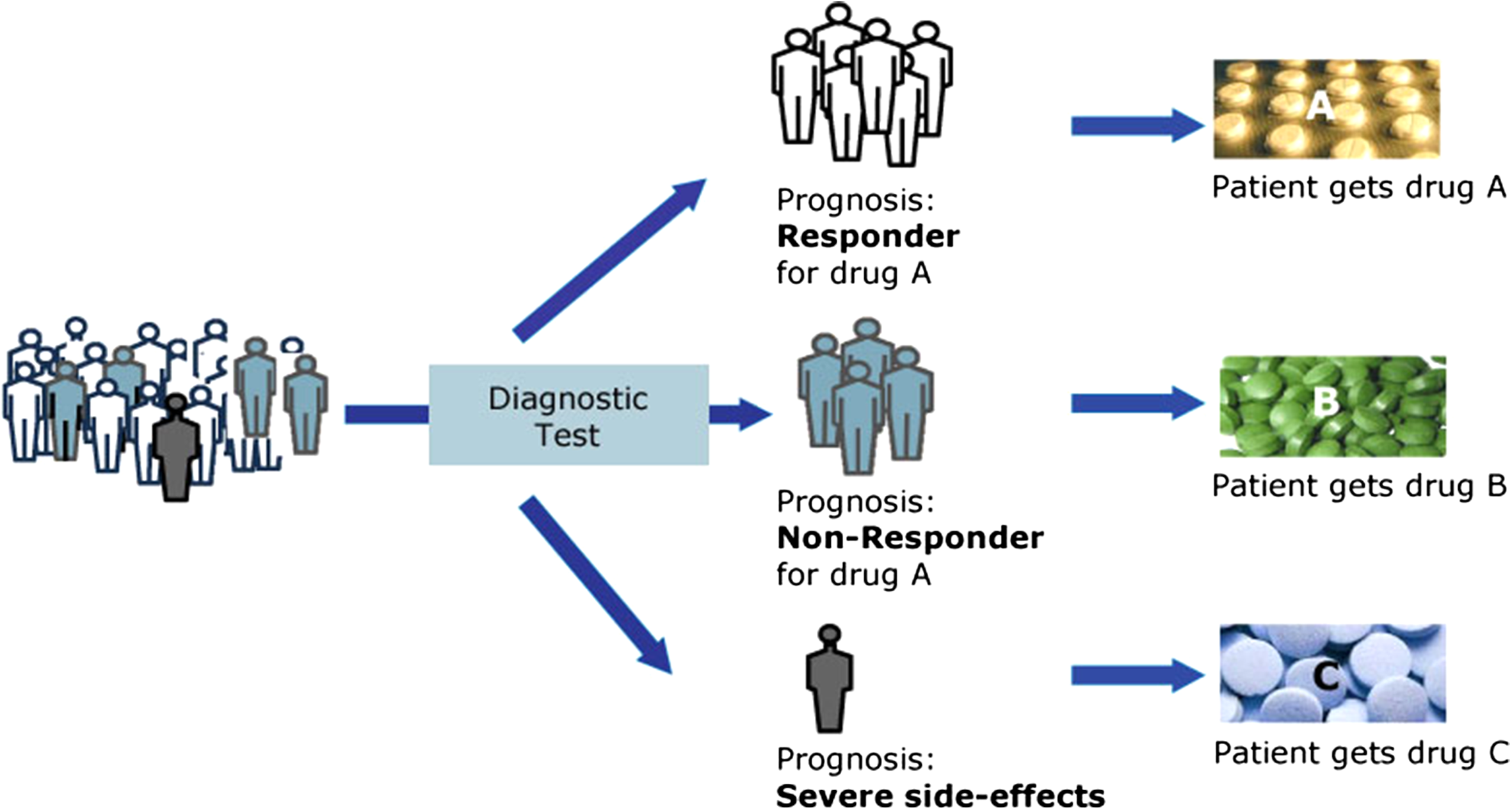

Supplement: Supplementary file 1 — Authors’ original file for figure 1 [file 11568_2011_3_MOESM1_ESM.tiff]

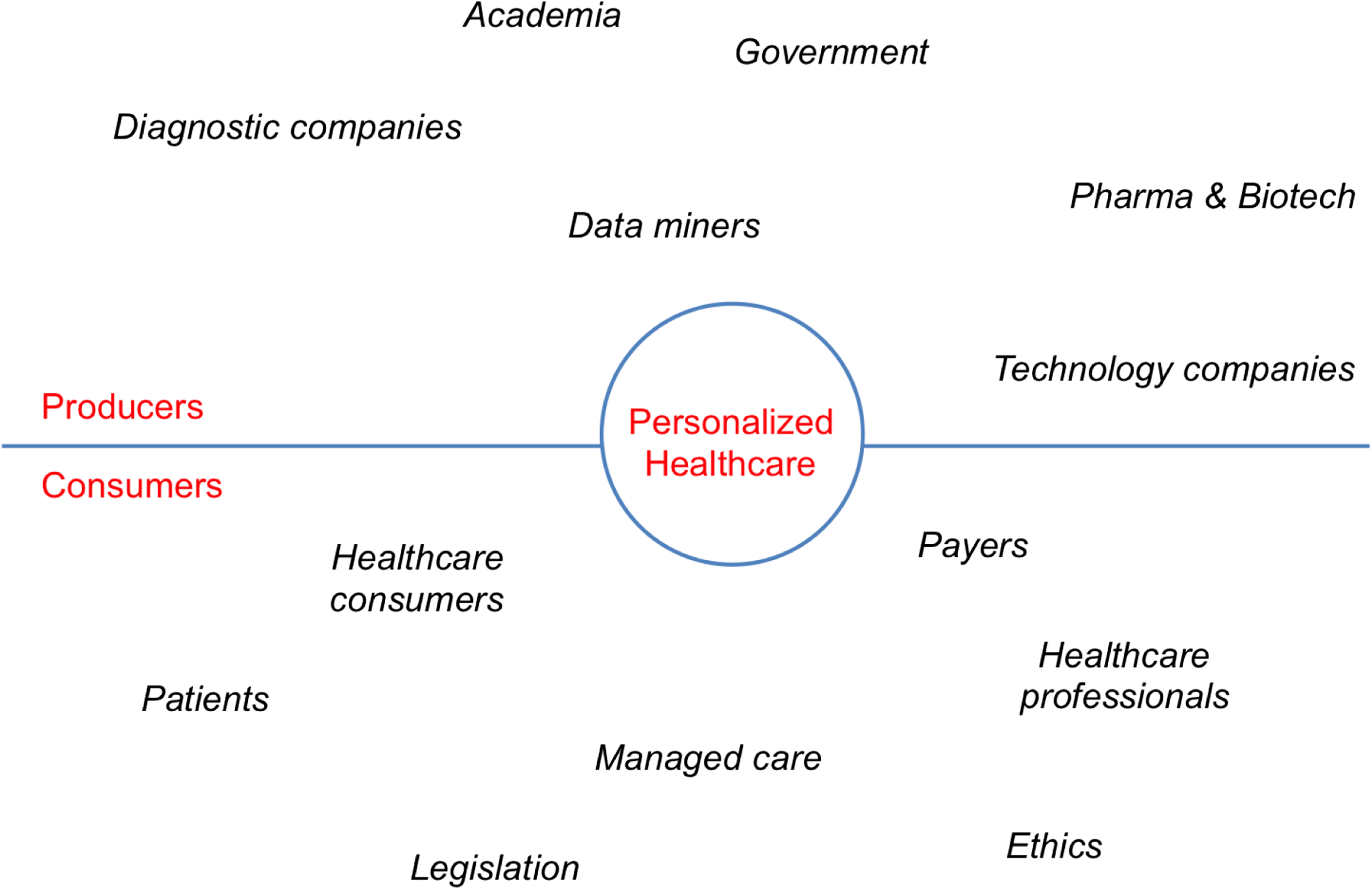

Supplement: Supplementary file 2 — Authors’ original file for figure 2 [file 11568_2011_3_MOESM2_ESM.tiff]
